# Clinical analyses of successful and previously failed intracytoplasmic sperm injection cycle parameters in patients with poor ovarian reserve

**DOI:** 10.4274/tjod.04382

**Published:** 2017-03-15

**Authors:** Tayfun Kutlu, Enis Özkaya, Pınar Kumru, Habibe Ayvacı, Belgin Devranoğlu, İlhan Sanverdi, Yavuz Şahin, Beyhan Sağlam, Ateş Karateke

**Affiliations:** 1 Zeynep Kamil Maternity and Children’s Health Training and Research Hospital, Clinic of Obstetrics and Gynecology, İstanbul, Turkey

**Keywords:** Poor responders, assisted reproduction, fertilization rate, gonadotropin-releasing hormone antagonist

## Abstract

**Objective:**

To determine some major characteristic differences between two consecutive successful and unsuccessful intracytoplasmic sperm injection (ICSI) cycles in poor responders.

**Materials and Methods:**

Sixty women with poor ovarian response as determined using the Bologna criteria underwent ICSI cycles following an unsuccessful trial. Some parameters of both cycles including age, body mass index (BMI), serum follicle-stimulating hormone (FSH) and estradiol levels, antral follicle count, gonadotropin dosage, duration of stimulation, antagonist starting day, duration of antagonist administration, endometrial thickness at trigger day, number of total and fertilized oocytes, embryo transfer day, number of embryo cells, and fertilization rate were compared in the same patients to identify predictors of cycles with clinical pregnancy.

**Results:**

The mean age, BMI, serum FSH, estradiol concentrations, and antral follicle count were 35.9 years (range, 30-42 years), 25.9 kg/m^2^ (range, 18.4-33.5 kg/m^2^), 10.9 IU/mL (range, 7-13 IU/mL), 52.9 pg/mL (range, 11.6-75 pg/mL), and 4.7 (range, 2-10), respectively. A comparison of cycle characteristics showed a significantly higher total number of mature and fertilized oocytes in successful cycles. The fertilization rate was also significantly higher in cycles with clinical pregnancy. Early initiation of antagonist was shown to result in favorable outcomes. A comparison of embryo characteristics showed that transfer of higher-stage embryos and embryos with higher numbers of cells had a significant impact on cycle outcomes.

**Conclusion:**

Our comparison of parameters of failed and successful ICSI cycles in poor responders revealed significantly earlier antagonist initiation, higher total number of mature and fertilized oocytes, fertilization rate, and significantly higher stage of embryo development and cell numbers at transfer in cycles that resulted in clinical pregnancy.

## INTRODUCTION

Women prefer to postpone their pregnancy plans to older ages due to career concerns^(1,2,3)^. Consequently, these women face a high risk of failure at conceiving in spontaneous cycles and seek assisted reproduction, especially in industrialized countries. This demand from health care providers leads to growing numbers of difficult infertile patients to be treated through assisted reproductive techniques. Moreover, these patients fail to respond to standard stimulation protocols due to poor ovarian reserve. The European Society of Human Reproduction and Embryology introduced the Bologna criteria in 2011 in order to standardize the definition of poor ovarian response^(4)^. Some stimulation protocols modified with adjuvant therapies and increased gonadotropin doses were tried to obtain favorable outcomes in poor responders^(5)^. In a study on the association between the number of eggs and live birth, the number of eggs in in vitro fertilization (IVF) was accepted to be an indirect indicator for clinical success. Analyses of data revealed a non-linear relationship between the number of eggs and live birth rate following IVF treatment. The maximum live birth rates were obtained when approximately 15 eggs were retrieved^(6)^. Harvesting of 4-6 oocytes has been defined to be poor response^(7)^.

Several studies compared the cycle outcomes of gonadotropin-releasing hormone antagonist administration with flexible (according to follicular size) and fixed starting days. Meta-analyses on this issue revealed no statistically significant difference in pregnancy rate between flexible and fixed protocols. There was a statistically significant reduction in the amount of recombinant follicle-stimulating hormone (rFSH) with the flexible protocol^(8)^.

An inter-cycle variability of responses to gonadotropin stimulation was shown in a recently published study. In the study, the authors categorized patients according to the number of follicles on the day of human chorionic gonadotropin (hCG) administration as low (0-<6), normal (6-<18), and high (≥18), and showed that only 73.9% of patients remained in the same category after a new cycle^(9)^.

In this study, we tried to determine some major characteristic differences between two consecutive successful and unsuccessful cycles in patients with poor ovarian response.

## MATERIALS AND METHODS

After approval of the hospital ethics committee, this retrospective study was conducted from January 2014 to December 2014 in the IVF/intracytoplasmic sperm injection (ICSI) unit of Zeynep Kamil Women and Children’s Health Training and Research Hospital (approval number: 2014-183). A total of 60 women with a failed and subsequent successful ICSI cycle were retrospectively screened from the hospital database and cycles with and without successful outcomes were compared in terms of cycle characteristics. In order to determine the minimum number of subjects needed to be enrolled in this study in order to have sufficient statistical power, sample size calculation was performed before the study. The probability of a type-1 error (α), a difference being found although a difference does not exist, was calculated. We used an alpha cut-off of 5% (0.05). All participants had regular menstrual cycles, normal serum prolactin levels, and had not received hormone treatment in the last 3 months. The patients’ ages ranged from 30 to 42 years.

All patients underwent assisted reproductive technology treatment because of their previous poor response and/or poor ovarian reserves. At least two of the following three criteria had to be fulfilled to establish the definition of poor ovarian reserve: (1) advanced maternal age (>40 years) or any other risk factor for poor ovarian response; (2) a previous poor ovarian response (≤3 oocytes with a conventional stimulation protocol); (3) an abnormal ovarian reserve test [i.e. antral follicle count (AFC) less than 5-7 follicles or anti-Müllerian hormone below 0.5-1.1 ng/mL]. Women whose cycles did not reach the embryo transfer stage, and those with endometriosis, male factor infertility, and previous ovarian surgery were excluded from the study.

An antagonist protocol was used in all patients for both cycles. On the second day of the menstrual cycle, depending on the patient’s response, rFSH 300-450 IU were administered and follicular growth was monitored using transvaginal sonography. The dosage of rFSH was adjusted starting from day 5 of stimulation according to the ovarian response. Follicle monitorization was performed using two dimensional measurements of growing follicles and a calculation of the mean value at each visit.

Antagonist (Cetrorelix, Merck-Serono, Geneva, Switzerland) 0.25 mg/day was administered when the follicular size was 12-14 mm. After the follicular size reached 18 mm, recombinant hCG 250 µg was administered, and follicular puncture was performed after 34-36 hours. Next, the application of 8% vaginal progesterone gel twice/daily was started. The serum hCG level was measured 2 weeks later; if the serum hCG level was more than or equal to the normal level, ultrasonography was performed in the days following serum hCG level measurement to detect a fetal pulse to confirm clinical pregnancy.

Age, body mass index (BMI), serum FSH, estradiol, AFC, stimulation protocol, gonadotropin type and dosage, duration of stimulation, duration of antagonist administration, menstrual day at embryo transfer, embryo cell number, endometrial thickness at trigger day, total number of oocytes and fertilized oocytes and fertilization rates were compared between failed and successful consecutive trials with a maximum interval of 2 months.

Data were analyzed using SPSS 15.0 for Windows. The paired samples t-test was used to compare continuous variables between two separate cycles within the group. A p value <0.05 was accepted as statistically significant.

## RESULTS

The mean age, BMI, FSH, estradiol concentrations, AFC were 35.9 years (range, 30-42 years), 25.9 (range, 18.4-33.5 kg/m^2^), 10.9 IU/mL (range, 7-13 IU/mL), 52.9 (range, 11.6-75 pg/mL), 4.7 (range, 2-10) respectively (Table 1). A comparison of cycle characteristics showed a significantly higher total oocyte number and fertilized oocytes in successful cycles. The fertilization rate was also significantly higher in cycles with clinical pregnancy. Early initiation of antagonist was shown to result in favorable outcomes. A comparison of embryo characteristics showed that transfer of higher-stage embryos and embryos a higher number of cells had a significant impact on cycle outcomes. All comparisons of variables between the two cycles are summarized in Table 2.

## DISCUSSION

In this study, we assessed cycle characteristics in poor responders with and without successful clinical pregnancy such as age, BMI, serum FSH, estradiol, AFC, stimulation protocol, gonadotropin dosage, duration of stimulation, duration of antagonist administration, antagonist starting day, menstrual day at embryo transfer, embryo cell number, endometrial thickness at trigger day, number of total and fertilized oocyte and fertilization rates. Our data revealed that early initiation of antagonist, higher number of total, mature and fertilized oocyte number with higher fertilization rates and transferring significantly higher stage of embryo development and embryo cell numbers led to favorable outcomes in ICSI cycles.

Despite introduction of many protocols with different initial doses and types of gonadotropins, optimal management of patients who are poor responders is still a concern. In this study, we tried to identify characteristics of a successful cycle compared with a preceding failed cycle in the same patients with poor ovarian response. Most of the time, when responses to the standard dose of gonadotropins (225-300 IU) for a proper multifollicular growth fails, dose increments are attempted to obtain a better outcome. Therefore, high doses of gonadotropins were proposed for a couple of decades in poor responders. However, there are some conflicting data regarding the success of increased gonadotropin doses in the management of poor responders. Previous studies showed no enhanced ovarian response and/or better pregnancy rates when 450 U of increased doses of gonadotropins were used^(10,11,12)^. Furthermore, a recently published study indicated that an increased starting dose of FSH did not result in higher pregnancy rates, and outcomes were similar between groups with different gonadotropin starting doses (300 UI, 450 UI, and 600 UI) of gonadotropins with regard to retrieved oocytes, number of embryos obtained, and pregnancy rates^(13)^. In our study, the mean starting and total gonadotropin doses were similar between the two cycles. However, we found significantly earlier antagonist initiation in successful cycles. The modified early antagonist start protocol was introduced to improve cycle outcomes. It was claimed that improved mature oocyte yield could be enhanced through follicular synchronization. Additionally, significantly higher clinical pregnancy rates compared with the conventional antagonist protocol were reported^(14)^. Furthermore, delayed-start of antagonist protocol was proposed to result in favorable outcomes in terms of number of dominant follicles and mature oocytes retrieved, mature oocyte yield, and fertilization rates in poor responders. The authors concluded that this was the result of the promoting and synchronizing effect on follicle development without impairing oocyte developmental competence^(15)^. Besides a higher rate of fertilization, we also found significantly higher numbers of total, mature oocyte and earlier antagonist initiation in successful cycles. Especially in patients with poor ovarian reserve, the number of oocytes has a critical role for cycle outcome. Studies on this issue showed a significant relationship between the number of eggs and live birth in all age groups. A study proposed that the number of eggs in IVF was an indirect indicator for clinical success. The best outcome was obtained when approximately 15 eggs were retrieved^(6)^. However, a yield lower than 4-6 oocytes after stimulation has been considered to be poor response^(7)^. In our study, the mean total number of oocytes harvested during failed and successful cycles were 4.8 and 5.6, respectively. Although both results are within the range of the poor response definition, it seems that a minimal increase in total oocyte numbers with increased fertilization rates resulted in favorable outcomes.

Another factor is the fertilization rate, which was thought to be an indirect finding for oocyte quality and was shown to be a significant predictor for embryo implantation^(16)^. Some morphologic characteristics of oocytes, such as zona pellucida thickness, cytoplasm appearance, and polar bodies were investigated to select the best embryos to transfer and therefore further minimize the number of embryos transferred^(17,18,19,20)^. However, according to the accumulated data, most of these parameters had a minimal impact for this purpose^(21,22)^. Embryo grading systems were developed and found correlated with pregnancy outcomes. Despite their limitations, grading systems are the most commonly applied procedures in the selection of the most qualified embryo for transfer. Further embryo assessments focused on the zygote stage, evaluation of embryo behavior at early cleavage, and extended culture performed to day 5 showed improved pregnancy outcomes^(23,24)^. There are also some data at the molecular level for implantation prediction^(25)^. However, after adjustment of the aforementioned covariates, the fertilization rate was shown as a significant predictor for embryo implantation in a previous study^(16)^. As mentioned above, our data also showed significantly increased fertilization rates in cycles with clinical pregnancy.

The relationship between embryo quality and pregnancy rates has been shown in several studies^(26,27,28)^. Early cleaving 2-cell embryos have been shown to have higher pregnancy rates than patients without early-cleaving 2-cell embryos^(29)^, and furthermore, transfer of 4-cell embryos resulted in significantly higher implantation and pregnancy rates compared with transfers of 2 and 3-cell embryos. Additionally, cell number was found as the strongest predictor of pregnancy in day 3 embryos in a scoring system based on cell number, fragmentation, and other morphologic criteria deemed specific to day 3 embryos^(30,31,32)^. According to a Cochrane review, cumulative clinical pregnancy rates from cleavage stage resulted in higher clinical pregnancy rates than from blastocyst cycles^(33)^. Data showed a decreased overall embryo quality score in embryos that were kept in culture till day 3^(34)^. In our assisted reproductive technology clinic, we try to avoid keeping embryos in culture media for more than 3 days, except when the top quality embryo has not been determined. In majority of cases, we prefer 2 to 3-day embryo transfers, especially in the event of a low number of embryos. Our data showed that number of cells in 2 to 3-day embryo transfers had a critical role in ICSI cycles; the number of cells was significantly higher in cycles with clinical pregnancy (4.1 vs. 5.9, p<0.05).

## CONCLUSION

Early initiation of antagonist, higher number of total, mature oocyte yield, higher fertilization rates and transfer of embryos with higher number of cells were significant factors of successful outcomes in poor responders. Further research on this topic should be conducted with larger study populations to elaborate on the implications of our study, and to obtain more data to modify cycles for better results in poor responders.

## Figures and Tables

**Table 1:**
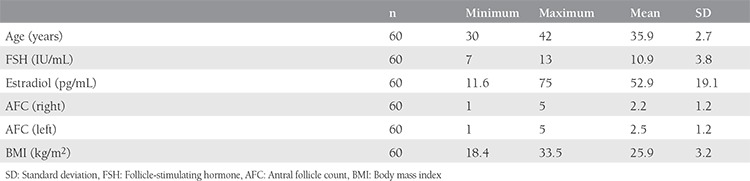
Summary of some demographic characteristics of study population

**Table 2:**
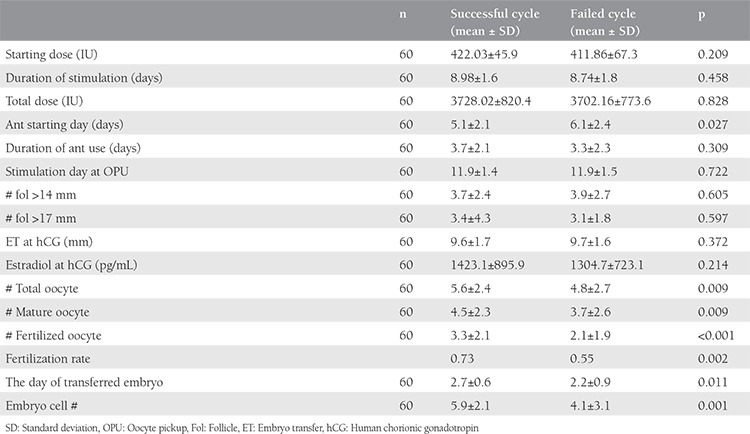
Comparison of some parameters of successful and preceding failed cycle in women with poor ovarian response
